# Asymmetric Protocols for Mode Pairing Quantum Key Distribution with Finite-Key Analysis

**DOI:** 10.3390/e27070737

**Published:** 2025-07-09

**Authors:** Zhenhua Li, Tianqi Dou, Yuheng Xie, Weiwen Kong, Yang Liu, Haiqiang Ma, Jianjun Tang

**Affiliations:** 1 China Telecom Research Institute, Beijing 102209, China; lizh84@chinatelecom.cn (Z.L.); xieyh@chinatelecom.cn (Y.X.); kongww1@chinatelecom.cn (W.K.); liuyang19@chinatelecom.cn (Y.L.); tangjj6@chinatelecom.cn (J.T.); 2 School of Physical Science and Technology, Beijing University of Posts and Telecommunications, Beijing 100876, China; 3 State Key Laboratory of Information Photonics and Optical Communications, Beijing University of Posts and Telecommunications, Beijing 100876, China

**Keywords:** quantum key distribution, mode pairing, particle swarm optimization, finite-key analysis, asymmetric protocols

## Abstract

The mode pairing quantum key distribution (MP-QKD) protocol has attracted considerable attention for its capability to ensure high secure key rates over long distances without requiring global phase locking. However, ensuring symmetric channels for the MP-QKD protocol is challenging in practical quantum communication networks. Previous studies on the asymmetric MP-QKD protocol have relied on ideal decoy state assumptions and infinite-key analysis, which are unattainable for real-world deployment. In this paper, we conduct a security analysis of the asymmetric MP-QKD protocol with the finite-key analysis, where we discard the previously impractical assumptions made in the decoy state method. Combined with statistical fluctuation analysis, we globally optimized the 10 independent parameters in the asymmetric MP-QKD protocol by employing our modified particle swarm optimization. Through further analysis, the simulation results demonstrate that our work achieves improved secure key rates and transmission distances compared to the strategy with additional attenuation. We further investigate the relationship between the intensities and probabilities of signal, decoy, and vacuum states with transmission distance, facilitating their more efficient deployment in future quantum networks.

## 1. Introduction

Classical encryption methods rely on computational complexity, which exposes them to the risk of being compromised by future advances in computability. In contrast, quantum key distribution (QKD) guarantees the absolute security of information transmission by leveraging the fundamental principles of quantum mechanics [1,2,3]. Since the inception of the first QKD protocol, namely the BB84 protocol [4], it has ignited a surge of research interest in the field of QKD. In QKD systems, single photons serve as the carriers of quantum keys, which cannot be amplified and are easily scattered or absorbed by the transmission channel. For the transmittance channel η, the secure key rate (SKR) cannot exceed the PLOB (Pirandola, Laurenza, Ottaviani, and Banchi) bound $R\leq -\log_{2}\left(1-\eta \right)$ [5], which represents the exact SKR limit for point-to-point QKD systems without quantum repeaters.

Twin-field (TF) QKD [6] utilizes single-photon interference for key generation, surpassing the PLOB bound, while also retaining immunity to detector-side attacks. Nevertheless, in order to maintain the coherence between distant quantum states, TF-QKD requires the deployment of global phase locking technology [7,8], leading to increased complexity and additional resource consumption within QKD systems. Fortunately, the recently proposed mode pairing (MP) QKD protocol [9,10] eliminates the requirement for global phase locking while also surpassing the repeaterless bound. Given its exceptional performance in laboratory [11,12] and field environments [13,14], the MP-QKD protocol has established itself as a robust candidate for the future deployment of quantum communication networks.

In scenarios involving geographical disparities (e.g., in star-shaped quantum communication networks [15]), or when the communicating parties (Alice and Bob) are situated in moving free spaces (e.g., drones, ships, satellites, etc.), challenges are posed in achieving symmetric communication with the central node, Charlie. Introducing additional losses to compensate for channel asymmetry is a solution, but it results in suboptimal SKR [16]. Previous analyses of the asymmetric MP-QKD protocol [17,18] required ideal decoy state assumptions and infinite-key, which are overly idealized and impractical. Thus, it is crucial to account for the finite-key size effects in the asymmetric MP-QKD protocol while considering practical decoy state deployment.

In this paper, we evaluate the performance of the MP-QKD protocol with asymmetric channels. Through employing the practical decoy state method and the universally composable framework, we analyzed the security of the asymmetric MP-QKD protocol with finite-key size. By employing our modified particle swarm optimization (PSO) algorithm, we globally optimized the 10 independent parameters of the decoy state MP-QKD protocol. Our modified PSO algorithm is more suitable for asymmetric MP-QKD, as it does not rely on the specific form or gradient information of the SKR function, demonstrating robust global search capabilities. Numerical simulation results show that when the distance difference between Alice–Charlie and Bob–Charlie is 50 km and 100 km, the SKRs with the asymmetric intensity strategy show a significant improvement compared to those obtained using the extra attenuation method, though they remain lower than that of the symmetric channel. We further present the relationship curves between signal and decoy state intensities (probabilities) and channel loss, and provide an analysis to explain the observed differences. Finally, we examine the effect of the maximum pairing interval on the performance of the asymmetric MP-QKD protocol.

## 2. Finite-Key Analysis for MP-QKD with Asymmetric Channels

Since the key size in practical implementations is not infinite, it is crucial to develop a framework that addresses the finite-key size effect. In previous studies on asymmetric MP-QKD [17,18], the key size was often assumed to be infinite, leading to overly idealized parameter estimation. In the following analysis, we extend the consideration from the infinite-key regime to the realistic finite-key scenario. At the end of the asymmetric MP-QKD protocol, Alice and Bob each share a key string, denoted as S and $S^{\prime }$, respectively. According to the universally composable framework [19], if these key strings satisfy both $\epsilon_{\mathrm{cor}}$-correct and $\epsilon_{\sec}$-secret, they are considered secure keys. $\epsilon_{\mathrm{cor}}$-correct refers to $\mathrm{Pr}\left(S\neq S^{\prime }\right)\leq \epsilon_{\mathrm{cor}}$. And $\epsilon_{\sec}$-secret requires satisfaction of the condition

(1) $$\frac{1}{2}{∥\rho_{AE}-U_{A}⊗\rho_{E}∥}_{1}\leq \epsilon_{\sec},$$

where ${∥\cdot ∥}_{1}$ denotes the trace norm, $\rho_{AE}$ is the density operator of the system of Alice and Eve, $U_{A}$ is the uniform mixture of all possible values of the bit string S, and $\rho_{E}$ is density operator of Eve’s system. In this way, the protocol with finite-key can be regarded as ϵ-secure, where $\epsilon =\epsilon_{\mathrm{cor}}+\epsilon_{\sec}$. In addition, we fix the security bound to $\epsilon =10^{-10}$.

In the following finite-key analysis of the asymmetric MP-QKD protocol, we assume the distances from Alice and Bob to Charlie are denoted as $L_{A}$ and $L_{B}$, where $L_{A}\leq L_{B}$ and $\Delta L=L_{B}-L_{A}$. In the *i*-th round, Alice prepares the coherent state $|e^{i\theta_{a}^{i}}\sqrt{k_{a}^{i}}\rangle$, where intensity $k_{a}^{i}$ is randomly selected from $\left\{\mu_{a},\nu_{a},o_{a}\right\}$ with probabilities $\left\{p_{\mu_{a}},p_{\nu_{a}},p_{o_{a}}\right\}$. And modulated phase $\theta_{a}^{i}$ is randomly chosen from $\left\{0,\frac{2\pi }{\Delta },\frac{4\pi }{\Delta },...,\frac{2\pi \left(\Delta -1\right)}{\Delta }\right\}$, where Δ is typically set to 16. In parallel, Bob randomly selects $k_{b}^{i}$ and $\theta_{b}^{i}$ to prepare the coherent state $|e^{i\theta_{b}^{i}}\sqrt{k_{b}^{i}}\rangle$ with probabilities $\left\{p_{\mu_{b}},p_{\nu_{b}},p_{o_{b}}\right\}$. It is important to note that in asymmetric channels, except for the case where $o_{a}=o_{b}=0$, the intensity and probability settings of Alice and Bob are unequal. Therefore, full parameter optimization is required to achieve the maximum SKR. After interference measurement, Charlie announces the measurement outcomes $D_{L},D_{R}\in \left\{0,1\right\}$ (0 represents “no click”, while 1 represents “click”). $D_{L}⊕D_{R}=1$ is considered as an effective detection. After *N* rounds, Alice and Bob pair each click with its immediate next neighbor within a maximum pairing interval *l* to form a successful pairing.

According to the intensities of the paired *i*-th and *j*-th rounds (j≤i+l), Alice (Bob) labels the “basis” as illustrated in Table 1.

Subsequently, Alice and Bob announce the basis, and the sum of the intensity pairs $\left(k_{a},k_{b}\right)=\left(k_{a}^{i}+k_{a}^{j},k_{b}^{i}+k_{b}^{j}\right)$ for each pairing. Subsequently, they perform the pair assignment based on Table 2. Notably, the 0-pair data are retained specifically for decoy-state parameter estimation.

For each *Z*-pair on location *i*, *j*, Alice (Bob) extracts a bit 0 when $k_{a}^{i}\neq k_{a}^{j}=o_{a}$ ($k_{b}^{j}\neq k_{b}^{i}=o_{b}$), and a bit 1 when $k_{a}^{j}\neq k_{a}^{i}=o_{a}$ ($k_{b}^{i}\neq k_{b}^{j}=o_{b}$). For each *X*-pair on location *i*, *j*, Alice (Bob) extracts a bit from the relative phase θabi−θabjθabi−θabjππmod2 and announces the alignment angle $\theta_{a\left(b\right)}=\left(\theta_{a\left(b\right)}^{i}-\theta_{a\left(b\right)}^{j}\right)\;\mathrm{mod}\;\pi$. Alice and Bob only retain the results with $\left|\theta_{a}-\theta_{b}\right|\leq \Delta$ or $\left|\theta_{a}-\theta_{b}\right|\geq \pi -\Delta$. In particular, when $\left|\theta_{a}-\theta_{b}\right|\geq \pi -\Delta$, Bob flips the bit. Thus, Alice (Bob) can then obtain the sifted key strings Z ($Z^{\prime }$) and X ($X^{\prime }$), derived, respectively, from the *Z*-pair and the *X*-pair. According to the decoy state method, Z ($Z^{\prime }$) is employed for generating secure keys, while X ($X^{\prime }$) is used to estimate the phase-error rate.

In finite-key scenarios, sifted keys may contain some errors. Here, error correction and privacy amplification are required to ensure both $\epsilon_{\mathrm{cor}}$-correct and $\epsilon_{\sec}$-secret of the keys. Alice sends $\lambda_{\mathrm{EC}}$ bits to Bob for performing key reconciliation, through which Bob computes an estimate ${\hat{Z}}^{\prime }$ of $Z^{\prime }$. Alice computes a hash of Z of length $\log_{2}\frac{2}{\epsilon_{\mathrm{cor}}}$ with a random ${\mathrm{universal}}_{2}$ hash function [20], which she sends to Bob together with the hash. If hash(${\hat{Z}}^{\prime }$) = hash(Z), this guarantees the $\epsilon_{\mathrm{cor}}$-correct of the keys; otherwise, the protocol aborts.

To ensure the security of final secure keys, Alice and Bob employ the privacy amplification based on the Quantum Leftover Hash Lemma [21,22], which offers a clear operational interpretation of smooth min-entropy. According to a random ${\mathrm{universal}}_{2}$ hash function is used to extract an $\epsilon_{\sec}$-secret key of length L from Z, where

(2) $$\epsilon_{\sec}=2\epsilon +\frac{1}{2}\sqrt{2^{L-H_{\min}^{\epsilon }\left(Z|E^{\prime }\right)}}.$$

Here, $E^{\prime }$ summarizes all information Eve learned about Z during the protocol. Based on a chain rule inequality [23], the smooth min-entropy $H_{\min}^{\epsilon }\left(Z|E^{\prime }\right)$ can be represented as

(3) $$H_{\min}^{\epsilon }\left(Z|E^{\prime }\right)\geq H_{\min}^{\epsilon }\left(Z|E\right)-\lambda_{EC}-\log_{2}\frac{2}{\epsilon_{\mathrm{cor}}},$$

where E is the information before error correction, $\lambda_{\mathrm{EC}}=fM_{\left(\mu ,\mu \right)}h\left(E_{\left(\mu ,\mu \right)}\right)$, *f* is the error correction efficiency, $M_{\left(\mu ,\mu \right)}$ represents the number of pairs $\left(\mu ,\mu \right)$ for *Z*-basis, and $E_{\left(\mu ,\mu \right)}$ denotes the corresponding bit error rate. Furthermore, the strings Z can be partitioned into two subsets: $Z_{11}$, which contains bits where both Alice and Bob each send a single photon, and $Z_{\mathrm{rest}}$, which comprises all remaining bits. Considering the smooth entropies from the uncertainty relation [22,24], we find that

(4) $$\begin{array}{cc} H_{\min}^{\epsilon }\left(Z|E\right)\geq & H_{\min}^{\bar{\epsilon }}\left(Z_{11}|Z_{\mathrm{rest}}E\right)+H_{\min}^{\epsilon^{\prime }}\left(Z_{\mathrm{rest}}|E\right)-2\log_{2}\frac{\sqrt{2}}{\hat{\epsilon }}, \end{array}$$

where $\epsilon =2\bar{\epsilon }+\epsilon^{\prime }+\hat{\epsilon }$ and $H_{\min}^{\epsilon^{\prime }}\left(Z_{\mathrm{rest}}|E\right)\geq 0$. It is evident that the single-photon components prepared in the *Z*- and *X*-bases are mutually unbiased. We denote $X_{11}$ (and $X_{11}^{\prime }$) as the bit string that Alice (and Bob) would have obtained had they performed measurements in the *X* basis instead of the *Z* basis. The quantity $H_{\min}^{\bar{\epsilon }}\left(Z_{11}|Z_{\mathrm{rest}}E\right)$ can be reformulated using the entropic uncertainty relation:

(5) $$\begin{array}{cc} H_{\min}^{\bar{\epsilon }}\left(Z_{11}|Z_{\mathrm{rest}}E\right) & \geq M_{11}^{Z}-H_{\max}^{\bar{\epsilon }}\left(X_{11}|X_{11}^{\prime }\right)\\ \; & \geq M_{11}^{Z}\left[1-h\left(e_{11}^{Z,\mathrm{ph}}\right)\right], \end{array}$$

where $M_{11}^{Z}$ denotes a lower bound on the length of $Z_{11}$, $h\left(e_{11}^{Z,\mathrm{ph}}\right)$ represents the number of bits required for Bob to use the bit string $X_{11}$ to reconstruct $X_{11}^{\prime }$, $e_{11}^{Z,\mathrm{ph}}$ is the phase-error rate associated with the single-photon pair events in the *Z*-basis, $h\left(x\right)=-x\log_{2}x-\left(1-x\right)\log_{2}\left(1-x\right)$.

We can set $\epsilon^{\prime }=0$ without compromising security. The total secrecy parameter is given by $\epsilon_{\sec}=2\hat{\epsilon }+4\bar{\epsilon }+\epsilon_{\mathrm{PA}},$ where $\bar{\epsilon }=\sqrt{\epsilon_{1}+\epsilon_{e}}$, with $\epsilon_{1}$ and $\epsilon_{e}$ representing the failure probabilities in estimating $M_{11}^{Z}$ and $e_{11}^{Z,\mathrm{ph}}$, respectively. The term $\epsilon_{\mathrm{PA}}$ denotes the failure probability associated with privacy amplification. By combining Equations (2)–(5), the final secure key length is

(6) $$L\leq M_{11}^{Z}\left[1-h\left(e_{11}^{Z,\mathrm{ph}}\right)\right]-\lambda_{\mathrm{EC}}-\log_{2}\frac{2}{\epsilon_{\mathrm{cor}}}-2\log_{2}\frac{1}{\sqrt{2}\hat{\epsilon }\epsilon_{\mathrm{PA}}}.$$

As long as the final key length satisfies Equation (6), the asymmetric MP-QKD protocol is ϵ-secure. The finite-key security analysis based on the entropy uncertainty relation method only requires consideration of the statistical fluctuations in observed quantities, without the need to account for additional information leakage. Here, the Chernoff bound [25] is employed to calculate the statistical fluctuations. Given an observed quantity χ, the upper $\bar{\chi }$ and lower $\underline{\chi }$ bounds of the expected value are given by,

(7) $$\begin{array}{cc} \bar{\chi }= & \chi +\beta +\sqrt{2\beta \chi +\beta^{2}},\\ \underline{\chi }= & \max\left\{\chi -\frac{\beta }{2}-\sqrt{2\beta \chi +\frac{\beta^{2}}{4}},0\right\}, \end{array}$$

where $\beta =\ln\frac{1}{\epsilon_{\mathrm{CB}}}$, and $\epsilon_{\mathrm{CB}}$ is the failure probability of the Chernoff bound.

## 3. Simulation and Discussion

Since the number of single-photon pair events $M_{11}^{Z}$ in the *Z*-basis and the phase-error rate associated with the single-photon pair events $e_{11}^{Z,\mathrm{ph}}$ in the *Z*-basis cannot be directly observed, through the decoy state method [12,26,27,28,29], we can discover the yield of *Z*-pair single photon pulse pairs

(8) $$y_{11}^{Z}=\frac{F^{L}-F^{U}}{a_{1}^{\nu_{a}}a_{1}^{\mu_{a}}\left(b_{1}^{\nu_{b}}b_{2}^{\mu_{b}}-b_{1}^{\mu_{b}}b_{2}^{\nu_{b}}\right)},$$

where

(9) $$\begin{array}{cc} F^{L}= & \frac{a_{1}^{\mu_{a}}b_{2}^{\mu_{b}}}{N_{\left(\nu_{a},\nu_{b}\right)}^{Z}}{\underline{n}}_{\left(\nu_{a},\nu_{b}\right)}^{Z}+\frac{a_{1}^{\nu_{a}}b_{2}^{\nu_{b}}a_{0}^{\mu_{a}}}{N_{\left(o_{a},\mu_{b}\right)}^{Z}}{\underline{n}}_{\left(o_{a},\mu_{b}\right)}^{Z}+\frac{a_{1}^{\nu_{a}}b_{2}^{\nu_{b}}b_{0}^{\mu_{b}}}{N_{\left(\mu_{a},o_{b}\right)}^{Z}}{\underline{n}}_{\left(\mu_{a},o_{b}\right)}^{Z}+\frac{a_{1}^{\mu_{a}}b_{2}^{\mu_{b}}a_{0}^{\nu_{a}}b_{0}^{\nu_{b}}-a_{1}^{\nu_{a}}b_{2}^{\nu_{b}}a_{0}^{\mu_{a}}b_{0}^{\mu_{b}}}{N_{\left(o_{a},o_{b}\right)}^{Z}}{\underline{n}}_{\left(o_{a},o_{b}\right)}^{Z},\\ F^{U}= & \frac{a_{1}^{\nu_{a}}b_{2}^{\nu_{b}}}{N_{\left(\mu_{a},\mu_{b}\right)}^{Z}}{\bar{n}}_{\left(\mu_{a},\mu_{b}\right)}^{Z}+\frac{a_{1}^{\mu_{a}}b_{2}^{\mu_{b}}a_{0}^{\nu_{a}}}{N_{\left(o_{a},\nu_{b}\right)}^{Z}}{\bar{n}}_{\left(o_{a},\nu_{b}\right)}^{Z}+\frac{a_{1}^{\mu_{a}}b_{2}^{\mu_{b}}b_{0}^{\nu_{b}}}{N_{\left(\nu_{a},o_{b}\right)}^{Z}}{\bar{n}}_{\left(\nu_{a},o_{b}\right)}^{Z}. \end{array}$$

Here, $a_{m}^{k_{a}}=\frac{{\left(k_{a}\right)}^{m}e^{-k_{a}}}{m!},b_{m}^{k_{b}}=\frac{{\left(k_{b}\right)}^{m}e^{-k_{b}}}{m!}$, ${\bar{n}}_{\left(k_{a},k_{b}\right)}^{Z}$ and ${\underline{n}}_{\left(k_{a},k_{b}\right)}^{Z}$ are the number of the effective detection for the intensity pairs $\left(k_{a},k_{b}\right)$ after the statistical fluctuations in the *Z*-pairs, $N_{\left(k_{a},k_{b}\right)}^{Z}$ is the expected number of pairs with $\left(k_{a},k_{b}\right)$. For simplicity, here we only use the *Z*-basis pairs with intensities $\left(\mu_{a},\mu_{b}\right)$ for key generation. Thus, $M_{11}^{Z}=N_{\left(\mu_{a},\mu_{b}\right)}^{Z}\mu_{a}\mu_{b}e^{-\mu_{a}-\mu_{b}}y_{11}^{Z}$.

The single-photon bit error rate of *X*-pair can be expressed as

(10) $$e_{11}^{X,\mathrm{bit}}=\frac{T^{U}-T^{L}}{a_{1}^{2\nu_{a}}b_{1}^{2\nu_{b}}y_{11}^{Z}},$$

where

(11) $$\begin{array}{cc} T^{U} & =\frac{1}{N_{\left(2\nu_{a},2\nu_{b}\right)}^{X}}{\bar{t}}_{\left(2\nu_{a},2\nu_{b}\right)}^{X}+\frac{a_{0}^{2\nu_{a}}b_{0}^{2\nu_{b}}}{N_{\left(2o_{a},2o_{b}\right)}^{X}}{\bar{t}}_{\left(2o_{a},2o_{b}\right)}^{X},\\ T^{L} & =\frac{a_{0}^{2\nu_{a}}}{N_{\left(0_{a},2\nu_{b}\right)}^{X}}{\underline{t}}_{\left(0_{a},2\nu_{b}\right)}^{X}+\frac{b_{0}^{2\nu_{b}}}{N_{\left(2\nu_{a},o_{b}\right)}^{X}}{\underline{t}}_{\left(2\nu_{a},o_{b}\right)}^{X}, \end{array}$$

and ${\bar{t}}_{\left(k_{a},k_{b}\right)}^{X}$ and ${\underline{t}}_{\left(k_{a},k_{b}\right)}^{X}$ are the error effective detection for the $\left(k_{a},k_{b}\right)$ after the statistical fluctuations in the *X*-pairs. Through a random-sampling theory without replacement [30,31,32], $e_{11}^{Z,\mathrm{ph}}$ can be written as

(12) $$e_{11}^{Z,\mathrm{ph}}\leq e_{11}^{X,\mathrm{bit}}+\Gamma \left(\xi_{ee},e_{11}^{X,\mathrm{bit}},M_{11}^{X},M_{11}^{Z}\right),$$

where

(13) $$\Gamma \left(a,b,c,d\right)=\sqrt{\frac{\left(c+d\right)\left(1-b\right)b}{cd}\ln\left(\frac{c+d}{2\pi cd\left(1-b\right)ba^{2}}\right)}.$$

$\xi_{ee}$ is the failure probability of random sampling without replacement. $M_{11}^{X}$ represent the number of single-photon pair events in the *X*-basis. After calculating $M_{11}^{Z}$ and $e_{11}^{Z,\mathrm{ph}}$, the secure key length for the asymmetric MP-QKD protocol under finite key analysis can be obtained by applying Equation (6). The SKR is defined as R=L/N, where *N* is the number of total pluses.

It should be clearly stated that although some studies have analyzed asymmetric MP-QKD, there remain certain limitations. For instance, Ref. [10] focuses solely on the advantage of asymmetric MP-QKD over TF-QKD, without examining the impact of channel asymmetry on the intensities and probabilities of decoy states. Additionally, Refs. [17,18] assumes that the decoy-state parameter estimation either meets idealized conditions or is performed with perfect accuracy, which is not representative of practical scenarios. For the gaps in their work, we have performed a thorough analysis addressing the effects of asymmetric channels on decoy-state parameters under practical conditions.

Here, we can consider the SKR as a function of the source parameters

(14) $$R\left(\vec{g}\right)=R\left(\mu_{a},\nu_{a},o_{a},p_{\mu_{a}},p_{\nu_{a}},p_{o_{a}},\mu_{b},\nu_{b},o_{b},p_{\mu_{b}},p_{\nu_{b}},p_{o_{b}}\right).$$

Given the uncertainty of the convex form of function *R*, we employ the modified PSO algorithm (detailed in Appendix A) for global optimization of the 10 parameters rather than the local search algorithm (LSA) [33,34,35]. The LSA is highly sensitive to the selection of the initial point, as a randomly chosen initial point can often result in an invalid or infeasible outcome. The modified PSO algorithm can optimize non-smooth and non-convex functions to search for the optimal $\vec{g}$ that maximizes *R*. It is especially effective for asymmetric MP-QKD because it does not depend on the specific form or gradient information of the SKR function, and it does not require an initial starting point, showing robust global search abilities. In comparison with the original PSO algorithm [36], the modified version demonstrates an improved balance between global exploration and local exploitation, strengthened particle coordination, and enhanced optimization efficiency and convergence speed. We note that Ref. [10] employs a genetic algorithm for parameter optimization, whereas our use of a modified PSO algorithm offers a simpler implementation with faster convergence and lower computational complexity, making it more suitable for practical deployment scenarios.

In the simulation, we employed an asymmetric intensity strategy to address the asymmetric channel: by deploying the PSO algorithm to optimize $\vec{g}$ for compensating the channel asymmetry and achieving the optimal SKR. The simulation formulas for the observed values in asymmetric MP-QKD are provided in Appendix B. Furthermore, we also compare this approach with the strategy of adding extra attenuation. It is important to note that in the simulation, we consider Alice’s and Bob’s source configurations as independent, satisfying

(15) $$\begin{array}{c} 0=o_{a\left(b\right)}<\nu_{a\left(b\right)}<\mu_{a\left(b\right)}<1,\\ p_{o_{a\left(b\right)}}+p_{\nu_{a\left(b\right)}}+p_{\mu_{a\left(b\right)}}=1,\\ 0\leq p_{\mu_{a\left(b\right)}},p_{o_{a\left(b\right)}},p_{\nu_{a\left(b\right)}}\leq 1 \end{array}$$

without imposing constraints such as $\eta_{a}\mu_{a}\approx \eta_{b}\mu_{b}$ (or $\eta_{a}v_{a}\approx \eta_{b}v_{b}$), where $\eta_{a}$ and $\eta_{b}$ are the channel transmittance of Alice and Bob. The formula $\eta_{a}\mu_{a}\approx \eta_{b}\mu_{b}$ (or $\eta_{a}v_{a}\approx \eta_{b}v_{b}$) is a rule of thumb on the ratio of intensities between Alice’s and Bob’s light [37]. Equation (15) imposes three constraints: the first line ensures that the intensity of the signal state is greater than that of the decoy state, which in turn is greater than that of the vacuum state; the second line enforces that the sum of the probabilities for the three states equals 1; and the third line guarantees that the individual probabilities for the signal, decoy, and vacuum states are all strictly between 0 and 1. The parameters employed for the numerical simulations are detailed in Table 3. Additionally, we set $\epsilon_{\mathrm{cor}}=\hat{\epsilon }=\epsilon_{\mathrm{PA}}=\xi_{ee}=\epsilon_{e}/12=\epsilon_{1}/7=3.28\times 10^{-23}$, where $\epsilon_{e}=11\epsilon_{\mathrm{CB}}+\xi_{ee}$, $\epsilon_{1}=7\epsilon_{\mathrm{CB}}$.

**Figure 1:**
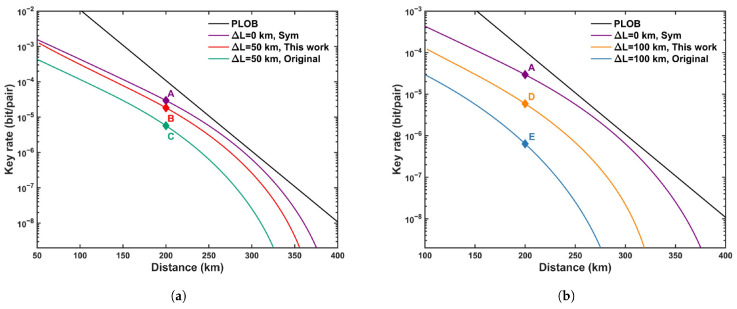
The optimized SKR versus the total distance ($L_{A}+L_{B}$) between Alice and Bob. The maximum pairing interval is fixed to 2000. The SKR is calculated for two cases, i.e., (**a**) a 50 km length difference between Alice and Bob, and (**b**) a 100 km length difference between Alice and Bob. Label ’Sym’ represents the symmetric case. Label ’This work’ refers to the asymmetric intensity method in this paper. Label ’Add att’ refers to the processing method, with added extra attenuation directly. The details of points A, B, C, D, and E are given in Table 4.

We plot the optimized SKR for the asymmetric scenario in Figure 1 and the detailed optimization results at a distance of 200 km in Table 4. From Figure 1, it is evident that the SKR of the asymmetric intensities strategy consistently outperforms that of the adding extra attenuation, for both ΔL=50 and ΔL=100. However, regardless of the strategy employed, the SKR does not achieve a level comparable to that of the symmetric channel case. The SKR for ΔL=100 experiences a significant decrease compared to ΔL=50, indicating that an increase in channel asymmetry leads to a reduction in the SKR. As evident from Table 4, the asymmetric intensities strategy exhibits a substantial increase of approximately an order of magnitude compared to the adding extra attenuation strategy when $L_{a}+L_{b}=200$ km. All data in Table 4 are optimized using the modified PSO algorithm. Furthermore, from points A, B, and D, it can be observed that Alice and Bob adjusted their intensities (probabilities) to compensate for the channel asymmetry. As ΔL increases, to achieve the optimal Hong–Ou–Mandel (HOM) interference effect, $\mu_{a}$ and $\nu_{a}$ continuously decrease, while $\mu_{b}$ and $\nu_{b}$ increase.

**Figure 2:**
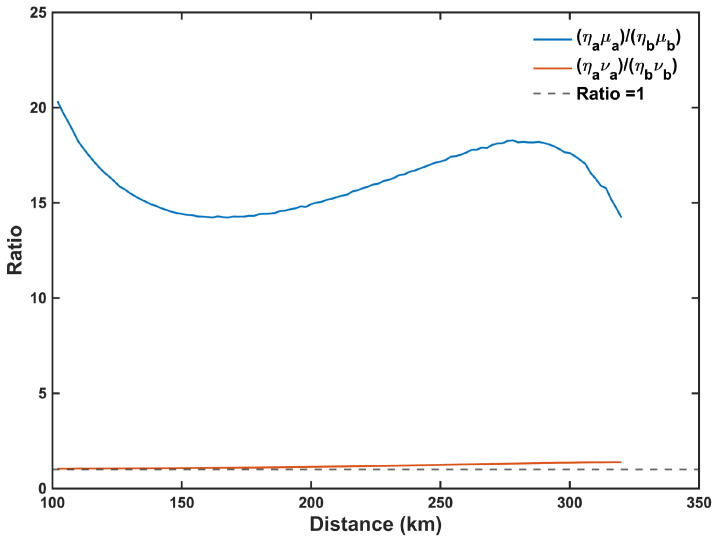
The optimized ratios $\frac{\eta_{a}\mu_{a}}{\eta_{b}\mu_{b}}$ and $\frac{\eta_{a}\nu_{a}}{\eta_{b}\nu_{b}}$ are plotted versus the total distance ($L_{A}+L_{B}$) between Alice and Bob, where ΔL=100 km and the maximum pairing interval l=2000.

In the asymmetric intensity strategy for MP-QKD, a primary concern is the optimal selection of weak coherent pulse intensities by Alice and Bob. While $\eta_{a}\mu_{a}\approx \eta_{b}\mu_{b}$ (or $\eta_{a}v_{a}\approx \eta_{b}v_{b}$) may seem natural, we do not impose this constraint in the modified PSO algorithm. Figure 2 depicts the variation of $\frac{\eta_{a}\mu_{a}}{\eta_{b}\mu_{b}}$ and $\frac{\eta_{a}\nu_{a}}{\eta_{b}\nu_{b}}$ for ΔL=100 km. It is evident that the magnitude of $\frac{\eta_{a}\nu_{a}}{\eta_{b}\nu_{b}}$ remains predominantly near 1, although some deviations occur as the distance increases. The decoy state $\nu_{a}$ ($\nu_{b}$) are primarily employed for estimating the phase error rate. The relationship $\eta_{a}v_{a}\approx \eta_{b}v_{b}$ helps maintain a balance in the photon intensities reaching Charlie, ensuring good HOM visibility and low error rates. However, this relationship is derived under the assumptions of the infinite-key size, an infinite number of decoy states, and the neglect of dark counts. Therefore, discarding the ideal assumption mentioned above, slight deviations in $\eta_{a}\nu_{a}$ and $\eta_{b}\nu_{b}$ are reasonable for the scenario depicted in Figure 2. Additionally, from Figure 2, it can be observed that the value of $\frac{\eta_{a}\mu_{a}}{\eta_{b}\mu_{b}}$ is significantly distant from 1, largely deviating from the condition $\eta_{a}\mu_{a}\approx \eta_{b}\mu_{b}$. The reason for this phenomenon is that the signal state is primarily used for key generation, where the intensity $\mu_{a}$ ($\mu_{b}$) not only impacts the quantum bit error rate but also influences the probability of sending single photons and error correction. The optimal selection of signal states $\mu_{a}$ ($\mu_{b}$) is a balance among quantum bit error rate, the probability of single-photon transmission, and error correction.

**Figure 3:**
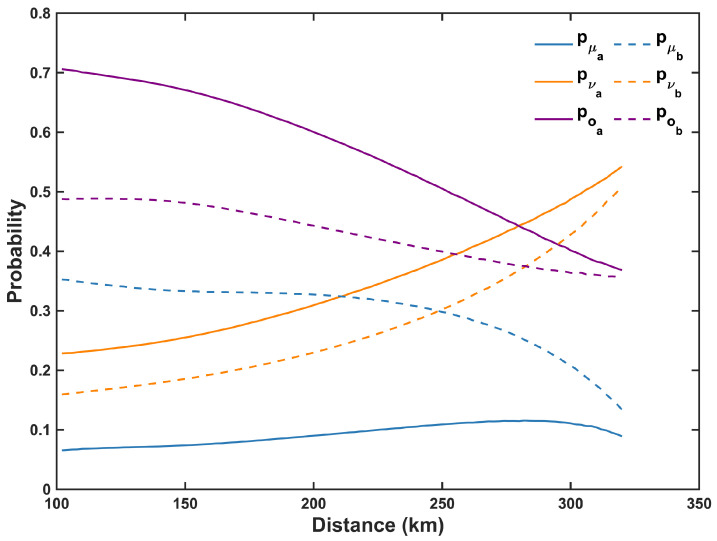
The optimized probabilities of the signal, decoy, and vacuum states with an asymmetric intensity strategy are plotted versus the total distance ($L_{A}+L_{B}$) between Alice and Bob, where ΔL=100 km and the maximum pairing interval l=2000.

Figure 3 illustrates the variations in the probabilities of the signal, decoy, and vacuum states with asymmetric intensity strategy as the distance increases, when ΔL=100 km. It can be observed that as the distance increases, the probability of the signal states remains relatively stable in most cases, while the probability of the decoy states notably increases. As the distance increases and losses grow, an inevitable increase in the probability of the decoy states is necessary to achieve better HOM interference effects and lower error rates. We also notice a relatively high proportion of vacuum states, attributable to their contribution in the pairing of the *Z*-basis, estimation of single-photon counts, and calculation of phase error rates. It is important to emphasize that for each optimization point in Figure 2 and Figure 3, the intensities and probabilities of the signal, decoy, and vacuum states strictly adhere to the constraints specified in Equation (15), while the counts and count rates are required to satisfy the physical validity conditions outlined in Equation (A1).

**Figure 4:**
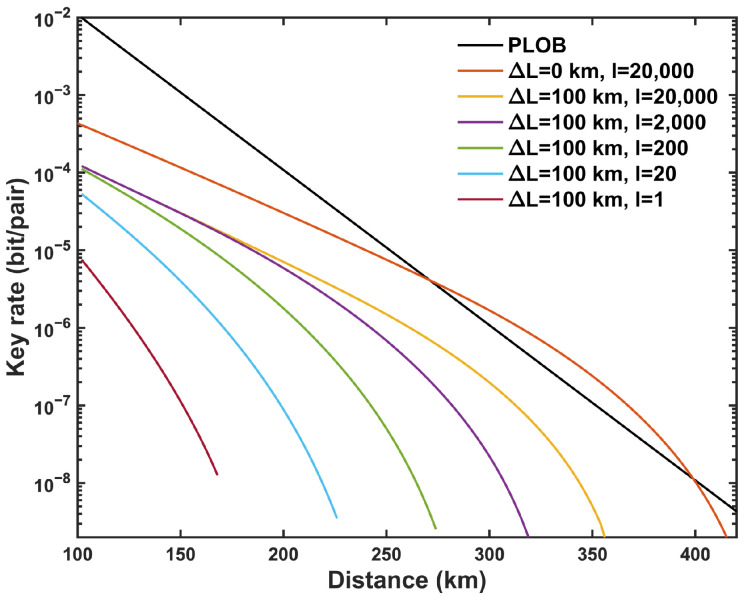
Optimized SKR with asymmetric intensity strategy versus the total distance ($L_{A}+L_{B}$) between Alice and Bob, at different maximal pairing intervals *l*.

**Figure 5:**
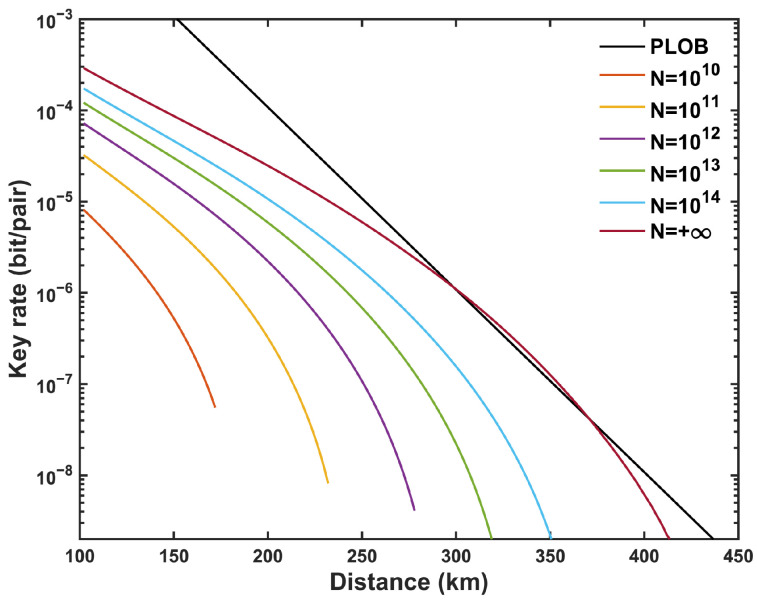
Optimized SKR with asymmetric intensity strategy versus the total distance ($L_{A}+L_{B}$) with different numbers of total pulses *N*, where l=2000 and ΔL=100 km.

Figure 4 demonstrates the influence of different maximum pairing intervals *l* on the SKR with asymmetric intensity strategy. With the increase in *l*, the SKR in the asymmetric scenario shows a significant improvement but remains below that of the symmetric case, unable to surpass the PLOB bound. Additionally, as shown in Figure 5, at long distances, the infinite-key scenario slightly surpasses the PLOB bound, representing the theoretical performance limit of MP-QKD in this regime. In contrast, under the finite-key condition, increasing the total number of pulses *N* makes it challenging to surpass the PLOB bound due to statistical fluctuations and finite-size constraints.

## 4. Conclusions

In this paper, we demonstrate the performance of the asymmetric MP-QKD protocol with finite-key analysis. By combining universal composability security analysis and practical decoy state methods, our work aligns more closely with real-world experimental conditions. In the PSO process for asymmetric MP-QKD, deploying an asymmetric intensity strategy results in a noticeable improvement in the SKR compared to adding extra attenuation and is more practical for real-world deployment. However, the SKR still remains lower than in the symmetric case. Simulation results indicate that the decoy state $\nu_{a}$ ($\nu_{b}$) is more susceptible to HOM interference, closely aligning with $\eta_{a}v_{a}\approx \eta_{b}v_{b}$. However, the signal state $\mu_{a}$ ($\mu_{b}$), influenced by multiple factors, significantly deviates from this assumption. Increasing the maximum pairing interval *l* can enhance the SKR in asymmetric MP-QKD, but it still falls significantly short compared to the symmetric case and remains unable to surpass the PLOB bound. Our research advances MP-QKD towards more practical network configurations and provides theoretical support for future MP-QKD quantum communication networks.

## Figures and Tables

**Table 1:** Basis assignment depending on intensities.

$k_{a(b)}^{j}$	$\mu_{a(b)}$	$\nu_{a(b)}$	$o_{a(b)}$
$k_{a(b)}^{i}$			
$\mu_{a(b)}$	*X*-basis	‘discard’	*Z*-basis
$\nu_{a(b)}$	‘discard’	*X*-basis	*Z*-basis
$o_{a(b)}$	*Z*-basis	*Z*-basis	‘0’-basis

**Table 2:** The pair assignment.

Alice	*Z*-Basis	*X*-Basis	‘0’-Basis
Bob			
*Z*-basis	*Z*-pair	‘discard’	*Z*-pair
*X*-basis	‘discard’	*X*-pair	*X*-pair
‘0’-basis	*Z*-pair	*X*-pair	‘0’-pair

**Table 3:** Parameters used in numerical simulation. $p_{d}$ and $\eta_{d}$ represent the dark counting rate per pulse and detection efficiency of SPD, respectively. α is the transmission fiber loss. *f* is the error correction efficiency. $e_{d}^{X}$ and $e_{d}^{Z}$ are the misalignment-error of the *X*-pair and *Z*-pair, respectively. *N* is the number of total pulses.

$p_{d}$	$\eta_{d}$	α	*f*	$e_{d}^{X}$	$e_{d}^{Z}$	*N*
$10^{-8}$	75%	0.2 dB/km	1.1	0.1	$10^{-6}$	$10^{13}$

**Table 4:** Examples of optimal parameters in Figure 1. The numerical values in the table presented here are rounded to three significant figures.

Point	$L_{A}+L_{B}$	ΔL	Strategy	$\mu_{a}$	$\nu_{a}$	$\mu_{b}$	$\nu_{b}$	$p_{\mu_{a}}$	$p_{\nu_{a}}$	$p_{o_{a}}$	$p_{\mu_{b}}$	$p_{\nu_{b}}$	$p_{o_{b}}$	*R* (Bit/Pulse)
♦ A	200 km	0 km	Sym	0.424	0.0213	0.424	0.0213	0.254	0.180	0.566	0.254	0.180	0.566	$2.95\times 10^{-5}$
♦ B	200 km	50 km	This work	0.216	0.00449	0.621	0.0376	0.170	0.229	0.601	0.305	0.192	0.503	$1.84\times 10^{-5}$
♦ C	200 km	50 km	Add att	0.492	0.0258	0.492	0.0258	0.271	0.220	0.509	0.271	0.220	0.509	$5.71\times 10^{-6}$
♦ D	200 km	100 km	This work	0.107	0.000624	0.718	0.0549	0.0902	0.309	0.6008	0.327	0.230	0.443	$5.89\times 10^{-6}$
♦ E	200 km	100 km	Add att	0.560	0.0321	0.560	0.0321	0.278	0.281	0.441	0.278	0.281	0.441	$6.37\times 10^{-7}$

## Data Availability

Dataset available on request from the authors.

## Appendix A. The Modified PSO Algorithm for Maximizing $R(\vec{g})$

**Algorithm 1:** The modified PSO Algorithm for Maximizing $R(\vec{g})$ with 10-Dimensional Variables

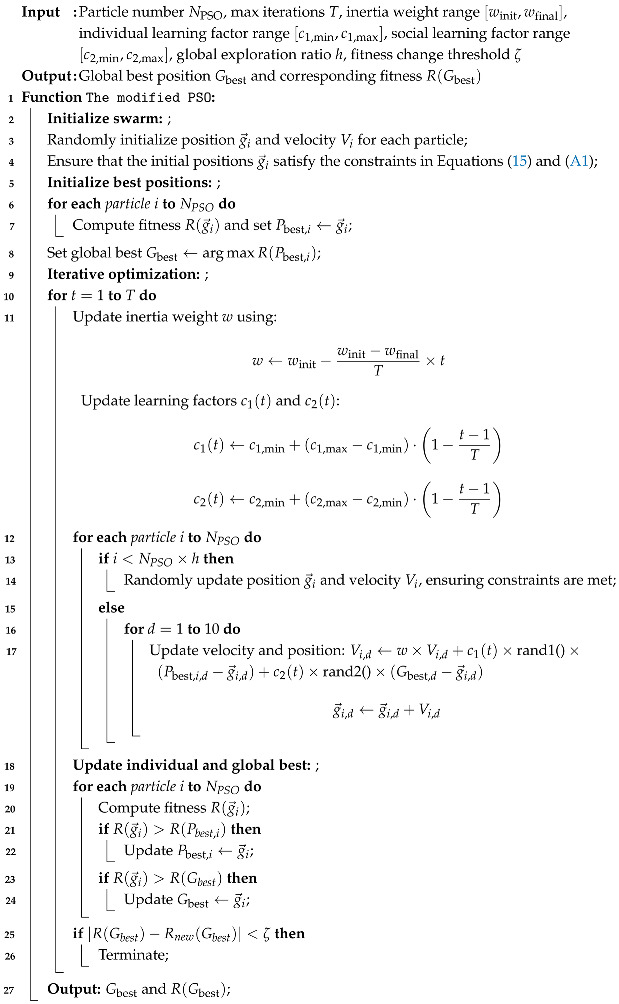

The PSO algorithm is a globally efficient and easily implementable optimization algorithm that reduces reliance on initial points through collaborative swarm behavior. It exhibits rapid convergence and is suitable for addressing a variety of complex multi-variable optimization problems. Considering the characteristics of the asymmetric MP-QKD protocol, we have modified the PSO algorithm as described in Algorithm 1. Lines 1–11 initialize the position, velocity, individual best, and global best of the particle swarm. It is reasonable and intuitive to expect that the particles, after initialization, should satisfy Equation (15). An often overlooked detail is that the intermediate parameters must also adhere to their physical significance, as shown below

(A1) $$\begin{array}{cc} \; & 0<n_{\left(k_{a},k_{b}\right)}^{Z},\;n_{\left(k_{a},k_{b}\right)}^{X},\;t_{\left(k_{a},k_{b}\right)}^{Z},\;t_{\left(k_{a},k_{b}\right)}^{X}\\ \; & 0<y_{11}^{Z},\frac{M_{\left(\mu ,\mu \right)}}{N}<1,\\ \; & 0<e_{11}^{Z,\mathrm{ph}},E_{\left(\mu ,\mu \right)}<0.5. \end{array}$$

Equation (A1), which guarantees that all counts are positive, all count rates fall between 0 and 1, and all error rates are greater than 0 but less than 0.5, aligns with the requirements for practical deployment. Compared to the original PSO algorithm [36], the modified version updates the inertia weight *w*, individual learning factor $c_{1}$, and social learning factor $c_{2}$ throughout the optimization process (Line 11). These updates improve the balance between global and local search, enhance particle coordination, and increase both efficiency and convergence speed. Lines 13–17 divide the particles into two groups: one group updates its position and velocity randomly, while the other follows the particle swarm optimization process. This effectively prevents the algorithm from getting trapped in local optima and enhances its global search capability. Lines 19–24 involve updating the individual best positions $P_{\mathrm{best},i}$, and the global best position $G_{\mathrm{best}}$. When the termination condition is met, the returned $G_{\mathrm{best}}$ and $R(G_{\mathrm{best}})$ represent the optimal decoy state settings and SKR. The modified PSO algorithm, independent of the specific form or gradient information of the SKR function *R*, demonstrates strong robustness. Its distinct advantage lies in its ability to handle complex, nonlinear problems, especially in high-dimensional spaces.

## Appendix B. Simulation Formulas for Asymmetric MP-QKD

The average response probability during each round *p* can be formulated as

(A2) $$p=\sum_{k_{a}^{i}k_{b}^{i}}p_{k_{a}^{i}}p_{k_{b}^{i}}q_{k_{a}^{i}k_{b}^{i}},$$

where $q_{k_{a}^{i}k_{b}^{i}}=2y\left[I_{0}\left(x\right)-y\right]$, $y=\left(1-p_{d}\right)e^{-\frac{k_{a}^{i}\eta_{a}+k_{b}^{i}\eta_{b}}{2}}$ and $x=\sqrt{\eta_{a}k_{a}^{i}\eta_{b}k_{b}^{i}}$. The expected pair number generated during each round can be expressed as

(A3) $$r_{p}=\left[\frac{1}{p\left[1-{\left(1-p\right)}^{l}\right]}+\frac{1}{p}\right].$$

For the *Z*-pair, where $\left(k_{a},k_{b}\right)\in \left\{\left(\mu_{a},\mu_{b}\right)\right.$, $\left(\mu_{a},\nu_{b}\right)$, $\left(\mu_{a},o_{b}\right)$, $\left(\nu_{a},\mu_{b}\right)$, $\left(o_{a},\mu_{b}\right)$, $\left(\nu_{a},\nu_{b}\right)$, $\left(\nu_{a},o_{b}\right)$, $\left(o_{a},\nu_{b}\right)$, $\left.\left(o_{a},o_{b}\right)\right\}$, the count of effective detections can be expressed as

(A4) $$n_{\left(k_{a},k_{b}\right)}^{Z}=\frac{Nr_{p}}{p^{2}}\sum_{\left(k_{a},k_{b}\right)}p_{k_{a}^{i}}p_{k_{a}^{j}}p_{k_{b}^{i}}p_{k_{b}^{j}}q_{k_{a}^{i}k_{b}^{i}}q_{k_{a}^{j}k_{b}^{j}}.$$

For $\left(k_{a},k_{b}\right)\in \left\{\left(\mu_{a},o_{b}\right)\right.$, $\left(o_{a},\mu_{b}\right)$, $\left(\nu_{a},o_{b}\right)$, $\left(o_{a},\nu_{b}\right)$, $\left.\left(o_{a},o_{b}\right)\right\}$ in the *Z*-pair, the number of error-effective detections is denoted by $t_{\left(k_{a},k_{b}\right),0}^{Z}=\frac{n_{\left(k_{a},k_{b}\right)}^{Z}}{2}$, while for $\left(k_{a},k_{b}\right)\in \left\{\left(\mu_{a},\mu_{b}\right)\right.,\left(\mu_{a},\nu_{b}\right),\left(\nu_{a},\mu_{b}\right),\left.\left(\nu_{a},\nu_{b}\right)\right\}$, it is denoted by

(A5) $$t_{\left(k_{a},k_{b}\right),0}^{Z}=\frac{Nr_{p}}{p^{2}}\sum_{\left(k_{a},k_{b}\right),k_{a}^{i}=k_{b}^{i}=0}p_{k_{a}^{i}}p_{k_{b}^{i}}p_{k_{a}^{j}}p_{k_{b}^{j}}q_{k_{a}^{i}k_{b}^{i}}q_{k_{a}^{j}k_{b}^{j}}+\frac{Nr_{p}}{p^{2}}\sum_{\left(k_{a},k_{b}\right),k_{a}^{j}=k_{b}^{j}=0}p_{k_{a}^{i}}p_{k_{b}^{i}}p_{k_{a}^{j}}p_{k_{b}^{j}}q_{k_{a}^{i}k_{b}^{i}}q_{k_{a}^{j}k_{b}^{j}}.$$

For the *X*-pair, where $\left(k_{a},k_{b}\right)\in \left\{\left(2\mu_{a},o_{b}\right)\right.$, $\left(o_{a},2\mu_{b}\right),\left(2\nu_{a},o_{b}\right)$, $\left.\left(o_{a},2\nu_{b}\right)\right\}$, the count of effective detections can be expressed as

(A6) $$n_{\left(k_{a},k_{b}\right)}^{X}=\frac{Nr_{p}}{p^{2}}\sum_{\left(k_{a},k_{b}\right)}p_{k_{a}^{i}}p_{k_{a}^{j}}p_{k_{b}^{i}}p_{k_{b}^{j}}q_{k_{a}^{i}k_{b}^{i}}q_{k_{a}^{j}k_{b}^{j}}.$$

The number of the error effective detections in this part can be represented as $t_{\left(k_{a},k_{b}\right),0}^{X}=\frac{n_{\left(k_{a},k_{b}\right)}^{X}}{2}$.

For $\left(k_{a},k_{b}\right)\in \left\{\left(2\mu_{a},2\mu_{b}\right)\right.$, $\left(2\mu_{a},2\nu_{b}\right)$, $\left(2\nu_{a},2\mu_{b}\right)$, $\left.\left(2\nu_{a},2\nu_{b}\right)\right\}$ in the *X*-pair, the count of effective detections can be expressed as

(A7) $$n_{\left(k_{a},k_{b}\right)}^{X}\approx \frac{Nr_{p}}{p^{2}}\frac{2\Delta }{\pi }p_{k_{a}^{i}}p_{k_{a}^{j}}p_{k_{b}^{i}}p_{k_{b}^{j}}\left(4y^{4}-8y^{3}I_{0}\left(x\right)+2y^{2}\left(I_{0}\left(x\sqrt{2-2\cos\Delta }\right)+I_{0}\left(x\sqrt{2+2\cos\Delta }\right)\right)\right),$$

where $I_{0}\left(\cdot \right)$ is the modified Bessel function of the first kind. The error-effective detections corresponding to this section can be expressed as

(A8) $$t_{\left(k_{a},k_{b}\right),0}^{X}\approx \frac{Nr_{p}}{p^{2}}\frac{2\Delta }{\pi }p_{k_{a}^{i}}p_{k_{a}^{j}}p_{k_{b}^{i}}p_{k_{b}^{j}}\left(2y^{4}-4y^{3}I_{0}\left(x\right)+2y^{2}I_{0}\left(x\sqrt{2-2\cos\Delta }\right)\right).$$

Considering the misalignment error, the error-effective detections of the *Z*-pair and *X*-pair can be rewritten as

(A9) $$\begin{array}{cc} t_{\left(k_{a},k_{b}\right)}^{Z} & =\left(1-e_{d}^{Z}\right)t_{\left(k_{a},k_{b}\right),0}^{Z}+e_{d}^{Z}\left(n_{\left(k_{a},k_{b}\right)}^{Z}-t_{\left(k_{a},k_{b}\right),0}^{Z}\right),\\ t_{\left(k_{a},k_{b}\right)}^{X} & =\left(1-e_{d}^{X}\right)t_{\left(k_{a},k_{b}\right),0}^{X}+e_{d}^{X}\left(n_{\left(k_{a},k_{b}\right)}^{X}-t_{\left(k_{a},k_{b}\right),0}^{X}\right), \end{array}$$

where $e_{d}^{Z}$ and $e_{d}^{X}$ is the misalignment-error of the *Z*-pair and *X*-pair, respectively.
